# Reduced emergent character of neural dynamics in patients with a disrupted connectome

**DOI:** 10.1016/j.neuroimage.2023.119926

**Published:** 2023-04-01

**Authors:** Andrea I. Luppi, Pedro A.M. Mediano, Fernando E. Rosas, Judith Allanson, John D. Pickard, Guy B. Williams, Michael M. Craig, Paola Finoia, Alexander R.D. Peattie, Peter Coppola, David K. Menon, Daniel Bor, Emmanuel A. Stamatakis

**Affiliations:** a Division of Anaesthesia, School of Clinical Medicine, University of Cambridge, UK; b Department of Clinical Neurosciences, University of Cambridge, Cambridge, UK; c Leverhulme Centre for the Future of Intelligence, Cambridge, UK; d The Alan Turing Institute, London, UK; e Department of Computing, Imperial College London, London, UK; f Department of Psychology, University of Cambridge, Cambridge, UK; g Department of Brain Science, Center for Psychedelic Research, Imperial College London, London, UK; h Data Science Institute, Imperial College London, London, UK; i Centre for Complexity Science, Imperial College London, London, UK; j Center for Eudaimonia and Human Flourishing, University of Oxford, Oxford, UK; k Department of Informatics, University of Sussex, Brighton, UK; l Department of Neurosciences, Addenbrooke's Hospital, Cambridge University Hospitals NHS Foundation, Cambridge, UK; m Wolfson Brain Imaging Centre, University of Cambridge, Cambridge, UK; n Department of Psychology, Queen Mary University of London, UK

**Keywords:** Whole-brain modelling, Network control theory, Hierarchy, Emergence, Information decomposition, Disorders of consciousness

## Abstract

High-level brain functions are widely believed to emerge from the orchestrated activity of multiple neural systems. However, lacking a formal definition and practical quantification of emergence for experimental data, neuroscientists have been unable to empirically test this long-standing conjecture. Here we investigate this fundamental question by leveraging a recently proposed framework known as “Integrated Information Decomposition,” which establishes a principled information-theoretic approach to operationalise and quantify emergence in dynamical systems — including the human brain. By analysing functional MRI data, our results show that the emergent and hierarchical character of neural dynamics is significantly diminished in chronically unresponsive patients suffering from severe brain injury. At a functional level, we demonstrate that emergence capacity is positively correlated with the extent of hierarchical organisation in brain activity. Furthermore, by combining computational approaches from network control theory and whole-brain biophysical modelling, we show that the reduced capacity for emergent and hierarchical dynamics in severely brain-injured patients can be mechanistically explained by disruptions in the patients’ structural connectome. Overall, our results suggest that chronic unresponsiveness resulting from severe brain injury may be related to structural impairment of the fundamental neural infrastructures required for brain dynamics to support emergence.

## Highlights

• Relationship between emergent dynamics, functional hierarchy, and controllability.
• DOC patients exhibit less hierarchical and emergent neural dynamics.
• DOC patients’ structural connectomes exhibit compromised modal controllability.
• Whole-brain model based on patient connectomes recapitulates functional alterations.

## 1 Introduction

Understanding how brain structure and function give rise to the functioning of the human mind is one of the major open challenges in contemporary neuroscience (Suárez et al., 2020). In addition to investigating how specific neuroanatomical regions contribute to brain function, it is also useful to study their dependence on highly distributed spatio-temporal patterns of collective activity arising from the complex interactions between multiple neural systems. At its core, this approach builds on the long-standing conjecture that mental activity may be an *emergent phenomenon* arising from the collective activity of neurons in the brain (Luppi et al., 2021; Mediano et al., 2022; Rosas et al., 2020; Turkheimer et al., 2019). Unfortunately, so far empirical investigations of this conjecture have been challenging, due at least in part to the absence of means to practically quantify emergence in experimental data. For the same reason, the potential relationship between emergence and functional and structural properties of the human brain still remains to be empirically investigated.

Thanks to recent technical breakthroughs, these questions can now be rigorously brought together under the same conceptual framework, and empirically investigated on neuroimaging data. By leveraging recent developments at the interface between information theory and dynamical systems, the *Integrated Information Decomposition* (ΦID) framework provides the means to conceptualise and quantify emergence in dynamical systems (Mediano et al., 2021). Specifically, it can be rigorously shown that a system exhibits *causal emergence* to the extent that its state as a whole provides information about its future state that cannot be obtained from the states of its individual components (Mediano et al., 2022; Rosas et al., 2020). In other words, causal emergence can be formally defined and mathematically quantified as *the causal (predictive) power of the macroscale, above and beyond the microscale effects* (Rosas et al., 2020). ΦID is widely applicable, offering rigorous methods to reason about and quantify causal emergence across a variety of different systems — from flocks of birds and Conway's celebrated Game of Life, to functional MRI measurements of human and non-human primate brain dynamics (Luppi et al., 2022c; Rosas et al., 2020).

By making emergence in the brain formally quantifiable, ΦID also makes it possible to contextualise how emergence relates to other fundamental properties of brain functional and structural organisation. Conceptually, emergence is deeply intertwined with the idea of *hierarchical organisation,* which (in its many possible conceptualisations (Hilgetag and Goulas, 2020)) is a fundamental principle in our contemporary understanding of the brain (Burt et al., 2018; Deco and Kringelbach, 2017; Demirtaş et al., 2019; Golesorkhi et al., 2022, 2021; Hansen et al., 2022, 2021; Margulies et al., 2016; Sydnor et al., 2021; Turkheimer et al., 2021). Thanks to ΦID, we are now in a position to characterise the empirical relationship between emergence and hierarchy in the activity the human brain.

Physiologically, the conditions for the dynamics of functional brain activity to exhibit hierarchical and potentially emergent properties are shaped by the structural connectome on which they unfold (Avena-Koenigsberger et al., 2017; Hagmann et al., 2008; Petersen and Sporns, 2015; Sporns, 2011; Suárez et al., 2020). Therefore, different physical configurations of the structural network may be expected to support different degrees of emergent or hierarchical dynamics. One attractive avenue to tackle the relationship between structural organisation and emergent dynamics is via the recent framework of *network control theory* (Gu et al., 2015), which studies how the organisation of a structural network shapes its ability to influence the functional dynamics that take place over it.

A unique opportunity to investigate how emergence is related to both functional and structural characteristics of the human brain comes from studying patients with chronic disorders of consciousness (DOCs) as a result of severe brain injury. Chronic DOCs involve permanent neuroanatomical damage, including disruption of the brain's structural connectivity and dynamics (Berlingeri et al., 2019; Cao et al., 2019, 2021; Cavaliere et al., 2015; Demertzi et al., 2019; Fernández-Espejo et al., 2011, 2012; Hannawi et al., 2015; Luppi et al., 2019, Luppi et al., 2021b; Luppi et al., 2021a; Wang et al., 2018; Zheng et al., 2017). In addition to providing a powerful avenue to relate brain organisation and (dys)function, this approach also addresses a pressing need to understand how the structural and functional brain reorganisation induced by DOC patients’ injuries prevent them from recovering (Claassen et al., 2021; Luppi et al., 2021). Therefore, in the present work we combine functional and diffusion MRI data to study brain function and structure in a cohort of 21 DOC patients and 18 healthy controls. We leverage ΦID and network control theory to investigate the relationship between emergence in brain dynamics, on one hand, and healthy and pathological aspects of the brain's structural and functional architecture, on the other.

Our main hypothesis was that emergent and hierarchical character of brain activity should be diminished in the brains of severely brain-injured unresponsive patients. Further, we hypothesised that the capability of these patients’ anatomical connectomes to control brain activity should be compromised as a result of their injury. Crucially, these hypotheses are tightly interconnected: emergence and hierarchy are two distinct but complementary ways of viewing the same dynamics, and a controllability shapes the repertoire of dynamics that the structural connectome can entertain. Therefore, as our final hypothesis we expect that causal emergence, functional hierarchy and structural controllability should be related to each other. To obtain mechanistic insights beyond pure correlation, we address this last hypothesis using *whole-brain computational models*, which simulate neurobiologically realistic brain dynamics based on different empirical connectomes (Cabral et al., 2017; Cofré et al., 2020; Deco and Kringelbach, 2014; Demirtaş et al., 2019; Kringelbach and Deco, 2020; Luppi et al., 2021; Shine et al., 2021; Wang et al., 2019). The model-generated dynamics can then be directly interrogated in terms of causal emergence via ΦID, through the same process as the empirical brain dynamics. This approach enables us to seek a mechanistic interpretation of our results. Through these convergent, multimodal investigations we shed light on how healthy and pathological brain structure influences brain dynamics.

## 2 Results

Here, we adopted the recently developed mathematical framework of Integrated Information Decomposition (ΦID) to quantify causal emergence in the dynamics of the human blood-oxygen-level dependant (BOLD) signal from fMRI data of N = 18 healthy controls and N = 21 DOC patients, further subdivided into N = 10 patients diagnosed with unresponsiveness wakefulness syndrome (UWS, also known as the vegetative state), and N = 11 patients in a minimally conscious state (MCS), who can occasionally exhibit behavioural signs consistent with transitory responsiveness. Through this powerful new approach to quantify emergence, we sought to investigate the fundamental connection between emergence and human consciousness, and how they both relate to relevant aspects of brain function (spatiotemporal hierarchy) and structure (network controllability).

### 2.1 Diminished emergence in the brain dynamics of DOC patients

**Fig. 1:**
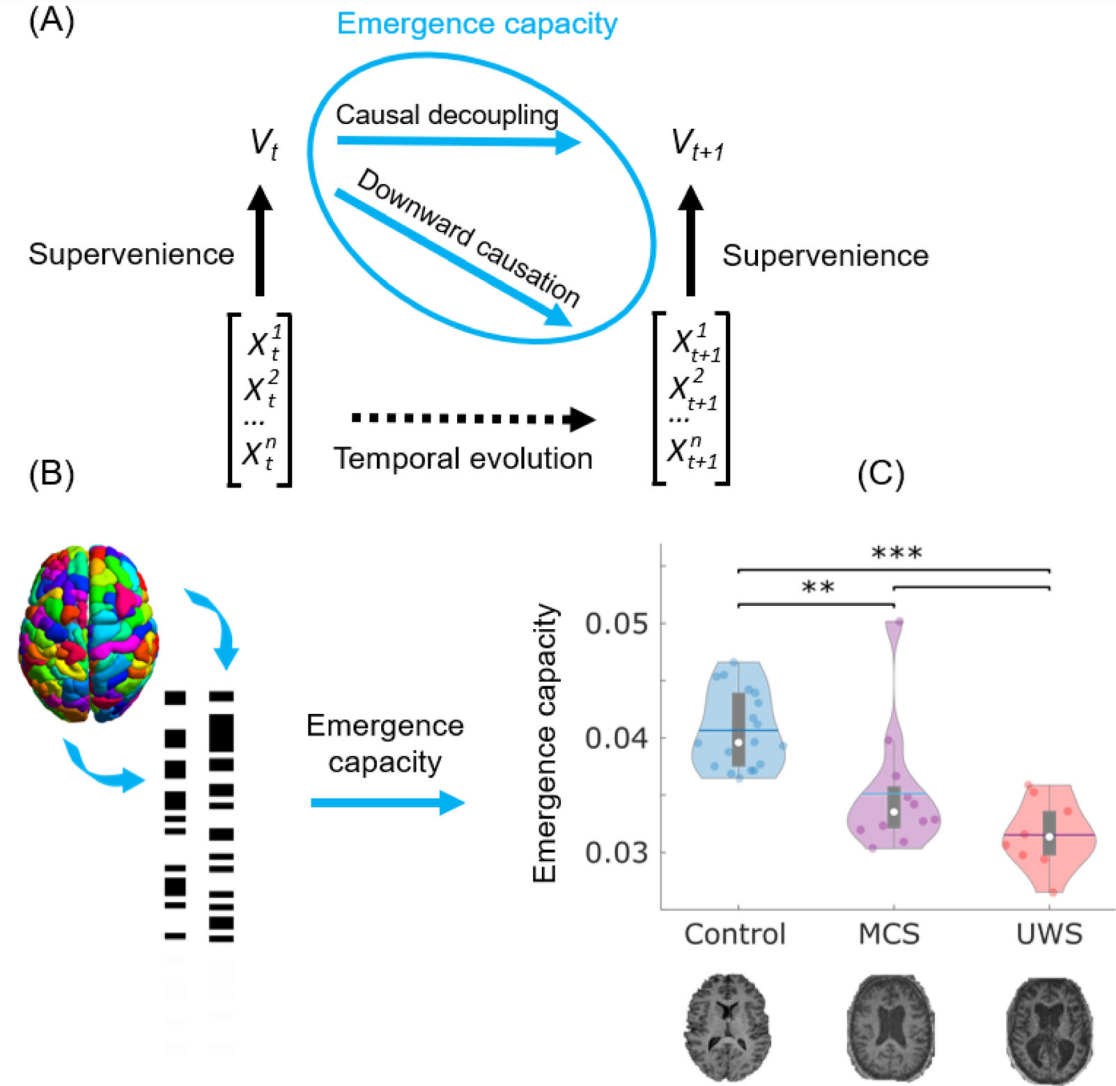
Causal emergence is diminished in the brain dynamics of DOC patients. (A) Relationship between emergence and supervenience. A macroscale feature Vt of a system is *supervenient* on the state of the system at time *t,* denoted by $X_{t}$, if $V_{t}$ is fully determined by $X_{t}$ (beyond the addition of noise), such that anything about $V_{t}$ that can be predicted from the system's previous state, $X_{t-1}$ can also be predicted from the system's current state, $X_{t}$. Then, a supervenient feature $V_{t}$  is said to be causally emergent if it has “unique” predictive power over the future evolution of the system $X_{t}$— in the sense of providing information about the dynamics of the system that cannot be found in any of the parts of the system when considered separately. The two components of emergence capacity are *causal decoupling*, the unique predictive power of $V_{t}$ on $V_{t+1}\;$corresponding to the system's macroscale influencing the macroscale's future;  and *downward causation*, the unique predictive power of  $V_{t}$ on $X_{t+1}\;$i.e. the macroscale influencing the microscale.  (B) The global emergence capacity of the human brain is obtained from Integrated Information Decomposition as the average emergence capacity (downward causation + causal decoupling) between each pair of discretised regional fMRI BOLD signals (Methods and Fig. S1). (C) Violin plots of each subject's emergence capacity by group. Data points represent subjects. White circle, median; centre line, mean; box limits, upper and lower quartiles; whiskers, 1.5x interquartile range. ** *p* < 0.01; *** *p* < 0.001, FDR-corrected. Here we used a time-step of 1 TR (2 s), the fastest available for our functional MRI data. No significant difference was observed when using a slower timescale of 4 TRs. We also show that analogous results are obtained using continuous (rather than discretised) signals (Fig. S3A), and using a different information-theoretic formalism (Methods and Fig. S3B), with UWS patients exhibiting significantly lower emergence capacity than healthy controls in both cases. We found that differences in emergence capacity can be attributed to downward causation (Fig. S4), rather than causal decoupling (all *p* > 0.05): that is, the overall difference in emergence capacity is primarily accounted for by the effects of the macroscale on the microscale.

To empirically investigate the hypothesis that the macroscale capacity for emergence is diminished in chronically unresponsive brain-injured patients, we adopted the account of causal emergence recently formalized by ΦID (Methods). Here, causal emergence has a specific technical meaning, that was recently formalized mathematically, and which is defined as follows (see Methods for further details). Given a system composed of multiple elements that co-evolve over time, we say that a macroscale feature *V_t_* is said to be causally emergent if it has “unique” predictive power over the future evolution of *X_t_* — in the sense of providing information about the dynamics of the system that cannot be found in any of the parts of the system when considered separately. Thus, supervenience is a relationship between the macroscale (for example, the shape of a flock of birds) and the microscale (the individual birds) at a particular point in time, whereas emergence pertains to the joint dynamics of the macro- and the microscale (Figs. 1 and S1) (Rosas et al., 2020). Crucially, ΦID allows one to define a quantity that upper-bounds the unique predictive power that any possible macroscopic feature could have. We refer to this quantity as “emergence capacity,” as it represents the ability of the system to host emergent features (see Methods).

Here, we employed ΦID to measure the capability for causal emergence of the coevolving activity of pairs of brain regions, based on their fMRI BOLD signals at rest (see Methods for details of how ΦID's information-theoretic quantities are computed). In other words, we quantify the capacity of pairs of regions to give rise to emergent behaviour together. By averaging the resulting estimates of emergence capacity across all pairs, we obtained an estimate of the global emergence capacity across the brain for each subject.

An analysis of variance revealed a significant effect of disorder severity (control, MCS or UWS) on the mean values of emergence capacity (F(2,37) = 26.08, *p <* 0.001), with subsequent post-hoc tests (corrected for multiple comparisons using the Benjamini-Hochberg procedure to control the false discovery rate (Benjamini and Hochberg, 1995)) indicating that healthy controls had significantly higher capacity for causal emergence than both MCS and UWS patients across brain regions - as well as a trend towards significance for the difference between patient groups (*p* = 0.072) (see Fig. 1 and Table S1). Thus, supporting our first hypothesis, we identified that lower causal emergence is observed in chronically unresponsive patients after severe brain injury. We further show that significant differences are also observed when considering the emergence capacity normalised by the total time-delayed mutual information in the system (Figure S2). Thus, both the total emergence capacity, and the proportion of information that is accounted for by emergence capacity, are diminished in DOC patients.

### 2.2 Compromised spatiotemporal brain hierarchy in DOC patients

**Fig. 2:**
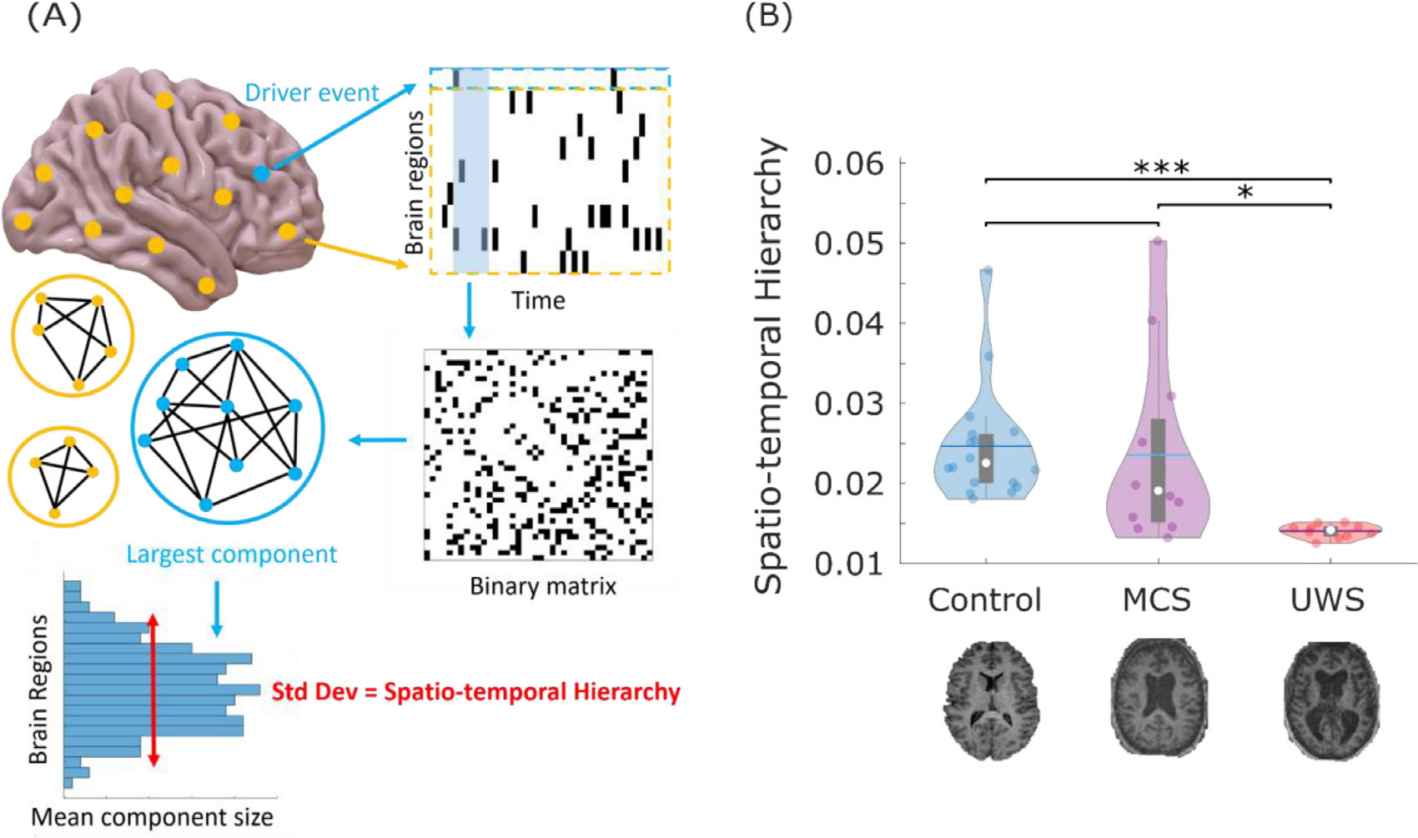
Spatio-temporal hierarchy of intrinsic-driven ignition is compromised in DOC patients. (A) intrinsic-driven ignition is obtained by identifying “driver events” (unusually high BOLD spontaneous activity; here, an event is defined to occur at a given region when its BOLD signal exhibits a Z-score larger than 1, following previous work (Deco et al., 2017; Deco and Kringelbach, 2017)), and measuring the magnitude of the concomitant activity occurring in the rest of the brain within a short time window (here, 4 TRs, approximately corresponding to the duration of the hemodynamic response function, following previous work (Deco et al., 2017; Deco and Kringelbach, 2017)). By the term “event” we refer to each regional occurrence of threshold-crossing; so if two regions cross the threshold within the same BOLD volume, then two events are occurring. The level of intrinsic-driven ignition is calculated as the size of the resulting largest connected component over a network linking regions that exhibit co-occurring events within the chosen time window. A measure of spatio-temporal hierarchy is obtained by calculating the variability across regions of their average IDI. (B) Violin plots of each subject's spatio-temporal hierarchy by group, showing that UWS patients exhibit diminished hierarchy compared with both healthy controls and MCS patients. Data points represent subjects. White circle, median; centre line, mean; box limits, upper and lower quartiles; whiskers, 1.5x interquartile range. * *p* < 0.05; ** *p* < 0.01, FDR-corrected.

Emergence is conceptually intertwined with another central concept in the modern neuroscientific literature: hierarchical organisation. In particular, recent theoretical and empirical work has shown that the global activation patterns that arise in response to spontaneous local activity induce a spatiotemporal hierarchy, whereby different regions vary in their capability to elicit spatially distributed neural activity over time — a phenomenon dubbed “intrinsic-driven ignition” (Fig. 2A) (Deco and Kringelbach, 2017). Crucially, in previous work, this spatio-temporal hierarchy (meaning, as a summary, the difference in elicited activity between the most and least influencing regions throughout the brain) was diminished during the transient unresponsiveness induced by both sleep and anaesthesia (Deco et al., 2017; Signorelli et al., 2020). Therefore, having identified an association between diminished emergence capacity and disorders of consciousness due to severe brain injury, we proceeded to test whether DOCs also induce a reduction in the spatio-temporal hierarchy of brain function.

Operationally, intrinsic-driven ignition (IDI) is obtained by identifying “driver events” of unusually high activity in spontaneous BOLD signals of each region and measuring the concomitant activity occurring in the rest of the brain. Importantly, regions generally vary in the extent of the ignition they typically elicit, and the spatial variability of the mean IDI across regions defines the brain's *spatio-temporal hierarchy*: when driver events in some regions are able to recruit a large fraction of the brain while events in others not at all, brain dynamics can be characterised as being highly hierarchical (Deco et al., 2017; Deco and Kringelbach, 2017). In other words, hierarchy is operationalised here in terms of a steeper difference of ranking between regions (in terms of their capacity to elicit broad ignition): when regions are all near-equal, there is low hierarchy, whereas when regions differ widely, then there is high hierarchy.

Results supported our hypothesis of diminished spatio-temporal hierarchy in the functional brain activity of DOC patients: an ANOVA revealed a significant effect of diagnosis on the spatiotemporal hierarchy of ignition (F(2,37) = 11.28, *p <* 0.001), with follow-up t-tests indicating that UWS patients exhibited reduced hierarchical organisation compared with both MCS patients and healthy controls (Fig. 2B and Table S2). Therefore, our results are in line with previous studies on sleep and anaesthesia (Deco et al., 2017; Signorelli et al., 2020), indicating that spatiotemporal hierarchy of the brain's intrinsic-driven ignition is compromised following the kind of severe brain injury that results in chronic disorders of consciousness.

To ensure the robustness of our results, we repeated our analyses pertaining to both emergence capacity and spatio-temporal hierarchy after controlling for mean framewise displacement as a covariate of no interest (Figure S5) and using a different parcellation size (129 ROIs; Fig. S6).

### 2.3 Reduced network controllability of the DOC connectome

Our results so far have shown that the brain activity of chronically unresponsive brain-injured patients compared to healthy controls is characterised by decreased causal emergence, and, possibly closely related to this, a diminished spatio-temporal hierarchy of brain dynamics. Crucially, however, brain dynamics are fundamentally shaped by the underlying structural connectome on which they unfold (Avena-Koenigsberger et al., 2017; Hagmann et al., 2008; Petersen and Sporns, 2015; Sporns, 2011; Suárez et al., 2020) - and indeed DOC patients often exhibit disrupted structural connectivity due to their injury, as well as subsequent complications and atrophy. To study how reductions in emergence and spatio-temporal hierarchy are related to brain structure we leverage principles of *network control theory*, which has recently become a prominent approach to investigate the relationship between the brain's network structure and its ability to support different kinds of functional dynamics (Betzel et al., 2016; Cornblath et al., 2018; Gu et al., 2015; Kim et al., 2018; Lynn and Bassett, 2019; Medaglia et al., 2017; Parker Singleton et al., 2021; Tang et al., 2020, 2017; Zarkali et al., 2020).

**Fig. 3:**
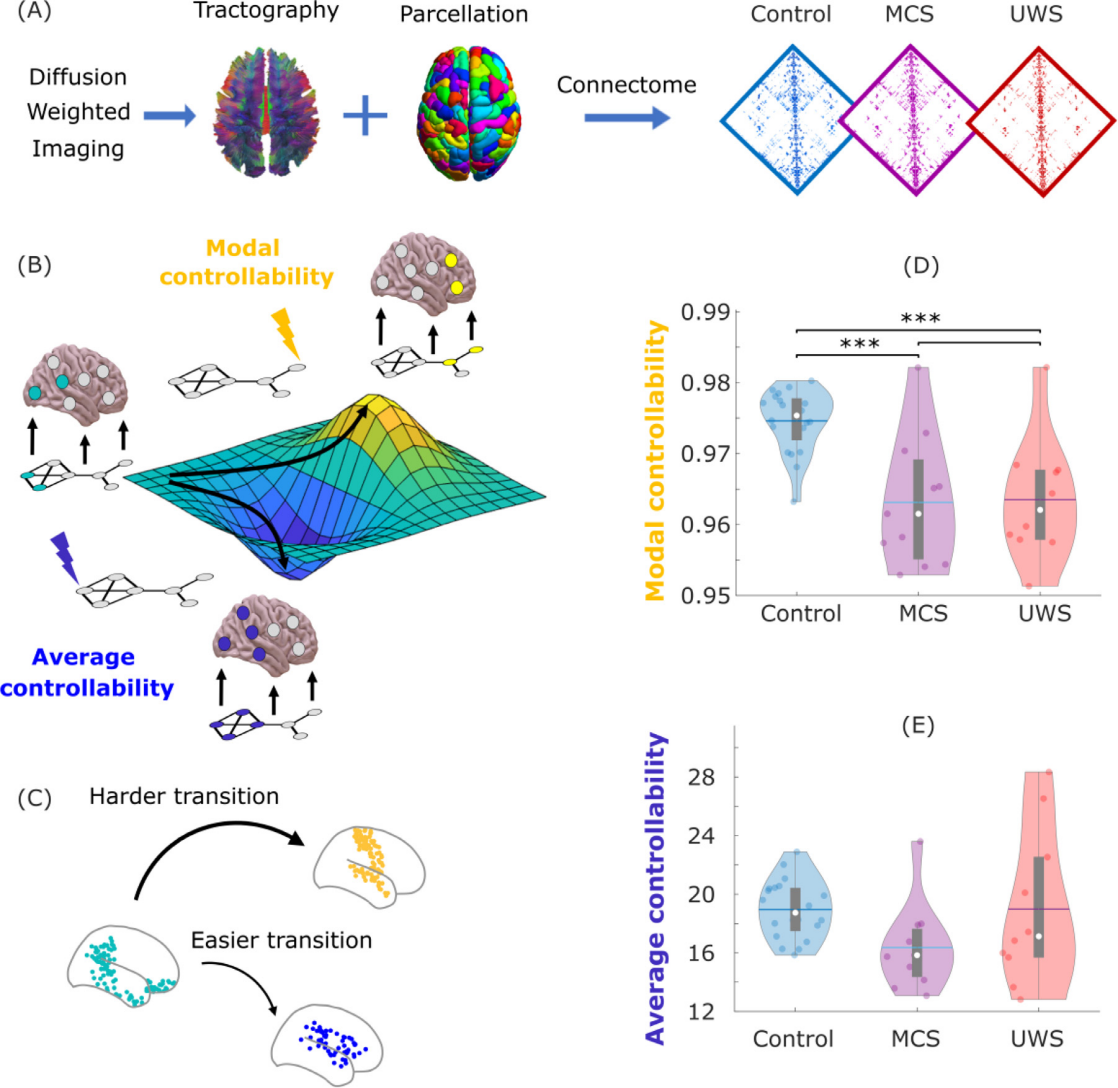
Reduced controllability of structural brain networks in DOC patients. (A) To obtain the structural connectome, diffusion weighted imaging (which measures the direction of water diffusion in the brain) is used to reconstruct white matter streamlines through tractography algorithms, obtaining a network representation of the physical connections between brain regions (here, *N* *=* 234 regions from the Lausanne atlas). The average structural networks for each group (control, MCS and UWS) are shown. (B) Functional brain activity (colored nodes are active, grey nodes are inactive) evolves through time over a fixed network structure (displayed below the brains). From a given starting configuration of activity (green), some alternative configurations are relatively easy to reach in the space of possible configurations (valley, in blue), whereas others are relatively difficult to achieve (peak, in yellow). To achieve a desired target configuration, input energy (represented by the lightning bolt icons) can be injected locally into the system, and it will spread to the rest of the system based on its network organisation. Average controllability quantifies the network's support for moving the system from an initial configuration of activity (green) to easy-to-reach configurations (blue), whereas modal controllability quantifies the network's support for moving the system to difficult-to-reach configurations of activity (yellow). (C) Example of easier and harder transitions from the literature (Karrer et al., 2020): starting from a baseline state corresponding to activation of the default mode network regions (green), previous work has shown that under the framework of linear network control theory it is easier to transition to activation of the limbic network regions (blue) than of the somatomotor network regions (yellow). (D) Global modal controllability is significantly reduced in DOC patients. Data points represent subjects. (E) Global average controllability across each group. White circle, median; center line, mean; box limits, upper and lower quartiles; whiskers, 1.5x interquartile range. *** *p* < 0.001, FDR-corrected.

Given a system of active elements (e.g., brain regions) interconnected by a network of structural connections (here, from the human connectome project), the organisation of the network's connections can be studied via control theory to determine how to intervene on the system to achieve a desired configuration of activity of its elements (Fig. 3A and B). Specifically, if energy is injected into the system via a particular node or set of nodes, it will spread to the rest of the system according to the network's connectivity, so that the activity of individual elements will be differently affected. As a consequence, a specific desired pattern of activity may be best achieved by intervening on some nodes rather than others. Nodes requiring comparatively less effort (i.e., smaller input energy) to achieve the same target configuration are said to be more *controllable*. Importantly, network control theory makes it possible to theoretically estimate controllability based on the structural network itself, without the need for physical interventions.

According to this formalism, different types of controllability can be defined depending on the type of desired outcomes. Here, we focus on two widely adopted and complementary notions: *average* and *modal controllability* (Methods) (Gu et al., 2015). Average controllability refers to the ability to steer the dynamics of the system towards configurations of activity that are relatively easy to reach, in the sense that they would require little energy to be injected into the system, because they are relatively close to the initial pattern of activity (Fig. 3B, blue). In contrast, modal controllability has been interpreted in the literature in terms of steering the dynamics of the system towards patterns of activity that are relatively difficult to reach, because they are very different from the current activity of the system (Fig. 3B, red). Considering the widespread alterations typically observed in DOC patients’ brain dynamics (Cai et al., 2020; Cao et al., 2021, 2019; Coppola et al., 2022; Demertzi et al., 2019; Huang et al., 2020; Luppi et al., 2019), our third hypothesis was that DOC patients should exhibit compromised controllability of their structural connectomes, reflecting a diminished capacity to control brain dynamics towards desired functional configurations.

We used diffusion MRI data to construct a network of structural connectivity for each subject in terms of the number of white matter streamlines connecting each pair of 234 cortical and subcortical regions (Cammoun et al., 2012) (Methods) (Fig. 3A). Based on each individual's structural connectome, we derived the average and modal controllability of each subject by taking the mean controllability across brain regions (Tang et al., 2017). Analysis of variance revealed a significant effect of diagnosis on whole-brain modal controllability between the three groups (F(2,36) = 14.50, *p <* 0.001) (Fig. 3C), while showing no significant differences in average controllability (F(2,36) = 2.79, *p* = 0.075) (Fig. 3D). Post-hoc pairwise t-tests (FDR-controlled) to explore the significant effect from the ANOVA indicated significantly higher modal controllability across brain regions for healthy controls than either MCS or UWS patients (Table S3). Analogous results were also obtained when using a different parcellation size (129 ROIs; Figure S7). Thus, our results indicate that the structural connectomes of DOC patients are significantly less suitable to steer their dynamics specifically towards hard-to-reach configurations - in line with existing results about the central importance of appropriate dynamics to support consciousness in humans and other mammals (Barttfeld et al., 2015; Demertzi et al., 2019; Gutierrez-Barragan et al., 2021; Luppi et al., 2019, 2020b; Uhrig et al., 2018)

### 2.4 Convergent evidence for structural-functional relationships

So far, we have demonstrated that the brains of chronically unresponsive brain-injured patients are characterized by reduced emergence and spatiotemporal hierarchy of brain activity, as well as structural network differences, specifically in terms of compromised modal controllability. This set of results raises the question of how exactly functional and structural deficits observed in these brain-injured patients are related with each other.

This section and the next will investigate the structure-function relationship via two convergent approaches: First, by correlating the functional (emergence, spatiotemporal hierarchy) and structural (modal controllability) measures that had exhibited significant differences between controls and DOC patients across all subjects (patients and controls); Second, to derive mechanistic insights beyond correlation, by using whole-brain computational modelling to generate biophysically realistic macroscale dynamics based on different connectomes, thereby illuminating how connectome structure shapes emergence and hierarchy. We report the results of these two investigations in turn below.

**Fig. 4:**
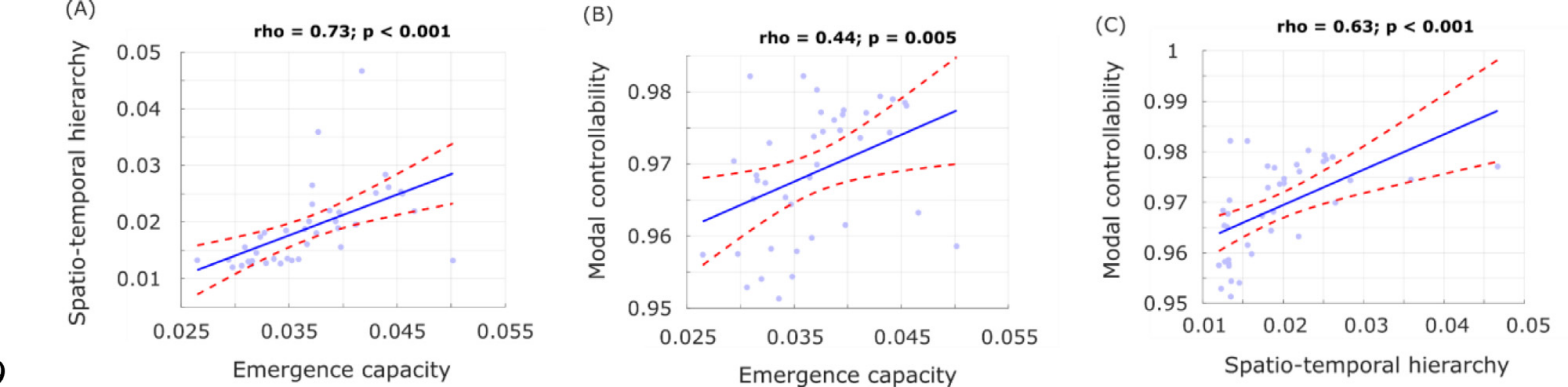
Functional and structural properties of the brain are correlated across subjects. Plots show Spearman's rank-based correlation tests between each pair of structural and functional measures that had exhibited significant differences across DOC patients and controls. Each data-point represents one subject (note that the two healthy controls and one DOC patient who did not have both structural and functional data were not included in this analysis).

Results of the correlation analysis supported our hypothesis, indicating significant positive values of Spearman correlation between all measures: causal emergence, spatiotemporal hierarchy, and overall modal controllability of the structural connectome (Fig. 4). These correlations have two key consequences: they support the theoretical link between emergence and spatiotemporal hierarchy, and they confirm our expectation that the presence of emergent and hierarchical dynamics in functional brain activity is related to controllability of the underlying structural connectome. In contrast, we did not observe significant differences across conditions in terms of the correlation between structural connectivity and pairwise emergence capacity (all *p* > 0.05).

### 2.5 Causal evidence for structure-function relationships from whole-brain computational models

Finally, we sought to determine whether the structural alterations observed in DOC patients may be part of the causal mechanism responsible for the observed functional deficits (emergence capacity and spatiotemporal hierarchy). To this end we employed whole-brain computational modelling, a powerful tool to investigate how macroscale neural dynamics emerge from the underlying anatomical connectivity (Cabral et al., 2017; Cofré et al., 2020; Deco and Kringelbach, 2014; Demirtaş et al., 2019; Kringelbach and Deco, 2020; Shine et al., 2021; Wang et al., 2019). These models represent regional macroscale activity in terms of two key ingredients: (i) a biophysical model of each region's local dynamics; and (ii) inter-regional anatomical connectivity. In particular, the neurobiologically plausible Dynamic Mean Field (DMF) model relies on a mean-field reduction to recapitulate the microscale neurophysiological properties of spiking neurons (Deco et al., 2013, 2014, 2018; Deco and Jirsa, 2012; Hansen et al., 2015; Herzog et al., 2020, 2022; Luppi et al., 2022b; Wong and Wang, 2006). Each cortical region is modelled as a macroscopic neural field comprising mutually coupled excitatory and inhibitory populations, and regions are then connected according to empirical anatomical connectivity obtained e.g., from diffusion weighted imaging (DWI) data (Deco et al., 2018, 2014, 2013; Deco and Jirsa, 2012; Hansen et al., 2015; Wong and Wang, 2006). The flexibility of this neurobiologically inspired whole-brain modelling makes it ideal to investigate how the anatomical connectivity of the brain shapes its macroscale neural dynamics (Cofré et al., 2020; Deco and Kringelbach, 2020; Shine et al., 2021).

**Fig. 5:**
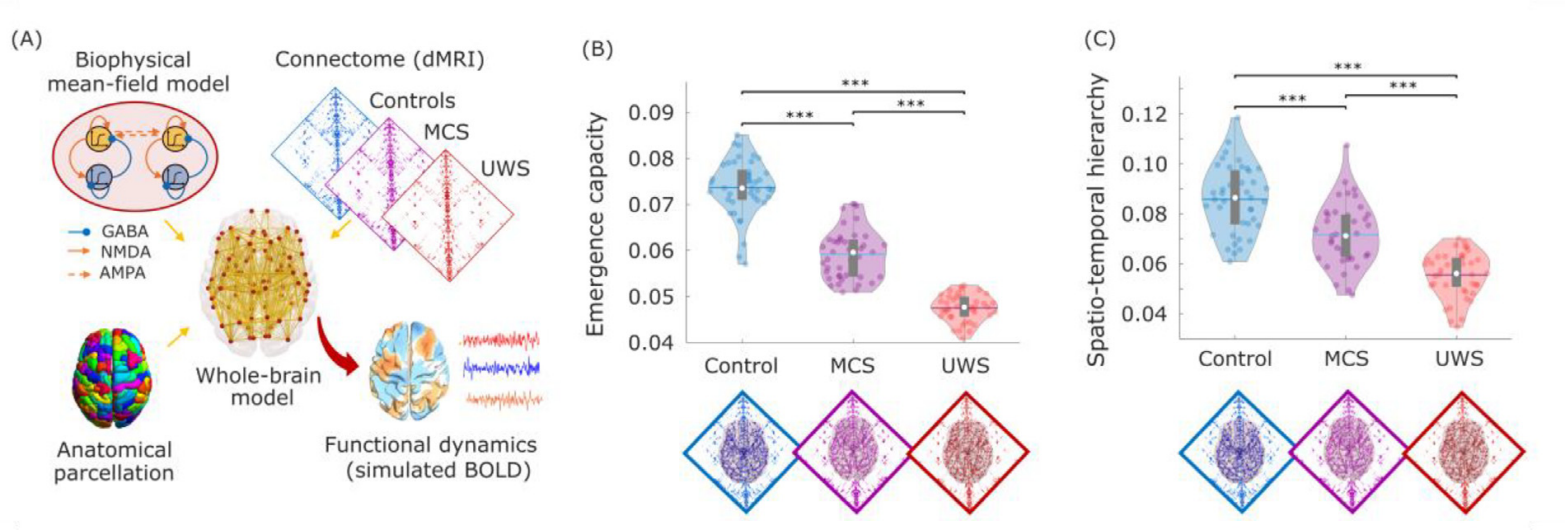
Whole-brain models informed by empirical connectomes replicate empirical changes in brain dynamics. (A) Overview of the whole-brain modelling approach to investigate structure-function relationships. The whole-brain model is based on local biophysical models of excitatory and inhibitory neuronal populations, corresponding to brain regions as defined by an anatomical parcellation, interconnected by a network of structural connections obtained from diffusion MRI from each group of subjects (healthy controls, MCS and UWS patients). The whole-brain model has one free parameter, the global coupling *G,* which is selected as the value just before the simulated firing rate becomes unstable. (B) Emergence capacity is highest in the dynamics simulated from control connectome, in line with empirical results. (C) Spatio-temporal hierarchical character is highest in the dynamics simulated from control connectome, in line with empirical results. Each data-point corresponds to one of 40 simulations obtained from each whole-brain model. White circle, median; centre line, mean; box limits, upper and lower quartiles; whiskers, 1.5x interquartile range. *** *p* < 0.001, FDR-corrected.

We fitted three whole-brain DMF models, each using a connectome obtained from combining the DTI of healthy controls, MCS patients, and UWS patients, respectively (Fig. 5A). The DMF model has one free parameter, the global coupling *G*; this parameter was selected separately for each model to lie just before the point where the simulated firing rate becomes unstable, which is typically where the model best reproduces empirical brain dynamics (Methods). Analyses performed on 40 simulations generated by each of these models replicated our main empirical findings, showing significant differences in emergence capacity and spatiotemporal hierarchy across each group – being highest in the model derived from healthy connectomes, and lowest in the model derived from UWS connectomes (Fig. 5B,C). We chose this data-agnostic tuning procedure to ensure that the results could be unequivocally attributed solely to the structural connectome. However, analogous results were also obtained when fitting the model *G* parameter to best match the empirical dynamics observed in the corresponding condition (Fig. S8). As with the empirical results, the results pertaining to global emergence capacity and spatio-temporal hierarchy could also be replicated using the 129-ROI parcellation (Fig. S9).

**Fig. 6:**
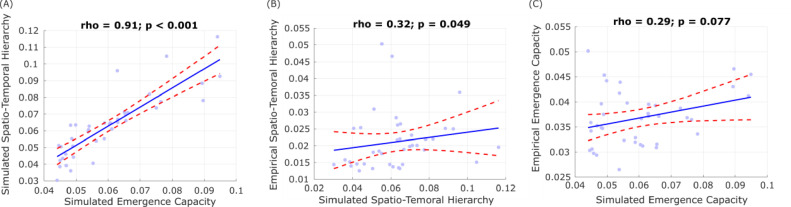
Correlation between empirical and simulated functional properties of the brain. Plots show Spearman's rank-based correlation tests between each pair of simulated functional measures (A), and between simulated and empirical measures (B,C).

Finally, we also used biophysical models based on each individual's structural connectome, to fit the functional connectivity dynamics of that individual. We then used the tuned individual models to simulate subject-wise BOLD signals, whose emergence capacity and ignition-driven spatio-temporal hierarchy we computed as for the empirical data. In accordance with previous results using a Hopf model (López-González et al., 2021), we observed lower *G* value for the DOC patients (mean = 2.41) than for the healthy controls (mean = 2.70; t(37) = 2.67, *p* = 0.011, Cohen's *d* = 0.84). Our modelling results show that the emergence capacity and ignition-driven spatio-temporal hierarchy were significantly and positively correlated in the simulated data (Fig. 6A), analogously to what we observed in empirical data. We also found that  ignition-driven spatio-temporal hierarchy was significantly and positively correlated between simulated and empirical data (Fig. 6B); though also positively correlated, emergence capacity narrowly failed to meet the standard threshold for statistical significance (Fig. 6C).

Overall, this computational modelling demonstrates that injury-induced changes in the structural connectome are sufficient to replicate the corresponding alterations in functional brain dynamics observed in chronically unresponsive patients (Fig. 5).

## 3 Discussion

The relationship between mind and emergence has been a recurrent open question in the philosophy of mind and cognitive science literature, but heated debates still persist — fostered by the lack of a practical operationalization of emergence applicable to empirical neuroimaging data (Turkheimer et al., 2019). Here, we present an empirical investigation of this long-standing question in neuroscience. We applied the recent framework of Integrated Information Decomposition to quantify the capacity of macroscale brain activity (from functional MRI recordings) to exhibit emergent phenomena.

Our results reveal that the capacity for causal emergence across the brain is significantly reduced following severe brain injury leading to chronic unresponsiveness. Subsequently, we investigated functional and structural correlates of emergence in the human brain. Functionally, our results show that the brains of chronically unresponsive patients are characterized by diminished hierarchical organization in the brain's ability to ignite distributed neural activity. To explore how these functional alterations are related to injury-induced changes in the brain's structural organisation, we used structural connectivity data obtained from diffusion-weighted MRI to examine differences in controllability of the structural connectome in DOC patients. Our investigation revealed that the organisation of DOC patients’ structural brain networks exhibits a consistent reduction in modal controllability, reflecting diminished structural support to achieve the desired functional configurations. In turn, this reduction in controllability of structural brain networks is associated with the observed functional reductions in emergence capacity and spatiotemporal hierarchy. Finally, a mechanistic relationship between structural and functional changes was confirmed by whole-brain computational modelling, which provided evidence that the kinds of structural alterations observed in chronically unresponsive patients may be sufficient to induce the corresponding deficits in functional emergence and hierarchy - both at the group-level and even at the single-subject level.

There is an important difference in interpretation between our measure of emergence capacity and traditional functional connectivity (FC): as a way to quantify co-fluctuations of BOLD activity over time, FC reflects a meaning of “integration-as-coupling”: the notion that if elements of a system behave similarly, they likely do so because of an underlying interaction existing between them. In contrast, emergence capacity reflects what we may refer to as “integration-as-complementarity”: being based on synergistic dynamics (Luppi et al., 2022c; Mediano et al., 2022), emergence capacity is high when the two elements interact in such a way that their joint contribution to their temporal evolution is more than the sum of their individual contributions. It is easy to see that integration-as-coupling (correlation) is maximised when one element is “enslaved” to be identical to the other, whereas in such a scenario integration-as-complementarity (emergence capacity) is nil - and both will be near-zero in the case of two unrelated processes. Therefore, FC and emergence capacity provide complementary perspectives and insights on the relationships that exist between elements of a system: here, the human brain.

It is noteworthy that of the two kinds of structural controllability investigated here, DOC patients exhibited global reductions in modal controllability. It has been argued that, under the specific parametrisation of controllability that is commonly adopted by investigations of average and modal controllability of the structural connectome (which is not the only possible one), modal controllability of the connectome may reflect the capability of a structural network to support transitions to functional configurations that are very different from the current one and therefore difficult to reach (Gu et al., 2015) (but see (Pasqualetti et al. 2019; Suweis et al. 2019; Tu et al. 2018)). We found that brains that are more modally controllable also exhibit greater hierarchical character (variability across regions) in terms of the capacity for local intrinsic events to ignite global propagation. We speculate that this ability to support global propagation of local activity may facilitate the presence of states of activity compatible with causally emergent dynamics - an intriguing possibility that opens several avenues for theoretical and empirical enquiry.

Together, our correlation between diminished functional properties and structural network alterations (here summarised in terms of diminished modal controllability), and our modelling results pertaining to diminished hierarchical integration and emergence capacity when using DOC patients’ connectomes, jointly suggest that DOC patients’ brain dynamics may suffer from insufficient structural support for transitions towards other states of activity.  By highlighting a critical role of structural network organisation in shaping the kind of dynamics that characterise consciousness, our results might contribute to explaining why DOC patients remain chronically unresponsive, unlike anaesthetised or asleep individuals, who also exhibit behavioural unresponsiveness and diminished hierarchical character of ignition, but only temporarily. Sleep and anaesthesia do not influence the connectome, which is therefore still capable in principle of supporting emergent dynamics. In contrast, the results of our whole-brain simulations indicate that the connectomes of DOC patients are less capable of supporting hierarchical and emergent brain dynamics - which seems to be critical for supporting consciousness and higher-order cognition, in line with recent proposals (Northoff et al., 2020).

### 3.1 Limitations and future directions

A number of limitations should be acknowledged when interpreting the results of the present study. Firstly, the account of emergence capacity adopted here is based on Integrated Information Decomposition, which is a recent development in the field of information theory and may be subject to further refinements as this field evolves (Mediano et al., 2021). In particular, although we have shown that our results are robust to the use of different operationalisations of information decomposition, methods for estimating ΦID in empirical data are not yet capable of accounting for all brain regions simultaneously, and therefore here we opted to use the average of all pairwise interactions as our quantification of global capacity to support causal emergence. Thus, we acknowledge that an important avenue for future work will be to extend our approach beyond pairwise interactions, and quantify causal emergence across larger groups of regions, up to the entire brain simultaneously, whether through theoretical developments or computational approximations. Indeed, recent advances in the related but complementary account of emergence proposed by Integrated Information Theory (Hoel et al., 2016; 2013; Klein and Hoel, 2020; Varley and Hoel, 2021) present promising avenues for future investigation (Luppi et al., 2021b). Similarly, “hierarchy” is a protean, multi-faceted concept in neuroscience (Golesorkhi et al., 2022; Hilgetag and Goulas, 2020), and different operationalisations may bear different relationships with emergence, which should be borne in mind when interpreting the present results.

Additionally, although here we capitalised on the availability of functional and diffusion MRI data in the same cohort of patients, future work may also seek to investigate emergence capacity from electrophysiological signals, which have higher temporal resolution and provide a more direct quantification of neuronal activity. This is especially relevant since we found that our results about emergence capacity depend on the timescale, and may be best characterised at a relatively short time-scale (by fMRI standards): extending the present results to electrophysiological data will enable a thorough investigation of the most discriminative timescale for the quantification of emergence capacity. It is also worth acknowledging that, although the network control framework adopted here for the analysis of structural connectivity has been recently used to model pathological and pharmacological changes in brain dynamics (Singleton et al., 2022; Zarkali et al., 2022; 2020), as well as pathological changes in regional metabolism (He et al., 2022), it still rests on assumptions about linearity (Gu et al., 2015; Lynn and Bassett, 2019). In contrast, the DMF model used is non-linear in character. These different modelling paradigms represent complementary avenues to interrogate the relationship between brain dynamics and the network structure of the underlying human connectome, despite their different assumptions and different levels of neurobiological fidelity. Nonetheless, there are several factors that point to the comparability of results from our controllability and whole-brain modelling analyses: First, the whole-brain model we use for our simulations (Dynamic Mean Field model; DMF) has been shown to be well approximated by linear dynamics (only small decrease in goodness-of-fit after linear approximation to the model, as per Deco et al. (2014)). Second, we replicated our results using emergence measures based on a linear-Gaussian approximation (Fig. S3). And finally, bridging between the two, convergent recent evidence suggests that empirical fMRI data may be adequately described by linear models (Nozari et al., 2020; Schulz et al., 2020). In future work, it would also be interesting to consider non-linear extensions of the tools from network control theory employed here, to converge onto a unified mathematical framework.

We also note that linear network control theory and nonlinear biophysical modelling are only two of a fast-growing number of methods for interrogating the relationship between brain network structure and function (Srivastava et al., 2020). Although we did not find statistically significant differences between conditions in terms of the correlation between pairwise emergence capacity and structural connectivity between regions, recent work has shown that complex changes in structure-function relationships can be identified in the brain of DOC patients through approaches such as dynamic functional connectivity (Demertzi et al., 2019; Huang et al., 2020) and eigenmode decomposition (Luppi et al., 2020b; Mortaheb et al., 2019; Panda et al., 2022). Such approaches have shown that abnormal structure-function coupling in DOC patients’ brains manifests in time-resolved and spatial frequency-resolved patterns. While time- and frequency-resolved extensions of Integrated Information Decomposition are ongoing, simultaneously accounting for all these complex relationships, and the complementary insights that they offer about healthy and pathological brain function, will be a key goal for future modelling work.

It is worth acknowledging that our results did not always identify statistically significant differences between healthy controls and MCS patients, or between MCS and UWS patients. We believe that this is likely due in part to the combination of our limited sample sizes and statistical stringency, since the larger sample sizes allowed by whole-brain modelling (40 for each group's connectome) provided statistically significant differences between each group. In this sense, biophysical modelling can serve the role of a data-augmentation tool, as recent work has also been exploring (Sanz Perl et al., 2020), to highlight effects of interest and isolate them from potential confounds such as physiological noise (Luppi et al., 2022a). However, it is important to bear in mind the caveat that such models are currently limited in the kind of information that they can take into account: as an example, cerebral metabolism is known to be compromised in DOC patients (Bodart et al., 2017; He et al., 2022b; Laureys et al., 1999; Sala et al., 2021), but not taken into account by current models. Likewise, we expect that explicitly incorporating information accounting for the differences between DOC aetiologies would increase these models’ clinical usefulness. Indeed, we openly acknowledge that our correlations between empirical and subject-level simulations were far from perfect: while the present results represent a powerful proof-of-concept for the ability of personalised biophysical models to reproduce *in silico* a number of empirically relevant functional measures based on structural alterations, such models are yet to reach their full potential for guiding personalised interventions for therapeutics.

Additionally, our correlation plots suggest that patients and controls may lie on a continuum in terms of these functional and structural characteristics of the brain, rather than occupying clearly defined categories. Thus, replication in a larger cohort may be warranted to shed light on the differences between patient subgroups. In this context, future work may investigate whether the structural connectomes of DOC patients who recover consciousness also show a corresponding recovery of modal controllability, and whether emergence from unconsciousness also corresponds to restored emergence in the brain's functional dynamics.

Relatedly, it will be important to obtain cross-modal validation of the present results: as a particularly relevant example, does greater emergence capacity of BOLD haemodynamics correlate with a higher Perturbational Complexity Index (Bodart et al., 2017; Casali et al., 2013; Casarotto et al., 2016; Rosanova et al., 2018; Sarasso et al., 2015)? This measure quantifies the complexity (information-richness) of the brain's electrophysiological responsiveness to causal intervention, in terms of TMS pulses, and it is arguably one of the most successful neuroimaging markers of consciousness, both in DOC patients and other perturbations of consciousness (anaesthesia, sleep) (Bodart et al., 2017; Casali et al., 2013; Casarotto et al., 2016; Rosanova et al., 2018; Sarasso et al., 2015). Therefore, it is natural to wonder whether the EEG patterns in response to TMS would also exhibit not only greater information content, but also a greater proportion of that information being accounted for in terms of emergence capacity, mirroring what is observed in the spontaneous BOLD signal. On the other hand, it is tempting to hypothesise that greater modal controllability of the DWI-based structural connectome may facilitate the spread of exogenous TMS perturbations and be reflected in more complex EEG patterns. It is also intriguing that the PCI was the very inspiration for Deco and Kringelbach (2017) development of the Intrinsic-Driven Ignition, which was intended as an endogenous counterpart of the PCI, applicable in the absence of exogenous perturbational data. Although we are not aware of PCI-IDI cross-modal comparison studies, such efforts would provide insights into the relationships between different ways of characterising brain dynamics, and their relationship with brain network architecture and its pathological reorganisation. Modelling efforts have also recently been undertaken to simulate PCI *in silico* (Goldman et al., 2021), and to evaluate the repercussions of different regional stimulation regimes (Deco et al., 2019; Sanz Perl et al., 2021): the extension of such models to fit both fMRI and EEG data will be an important step towards personalised medicine for DOC patients.

### 3.2 Conclusion

Overall, in the present work we combined a suite of cutting-edge computational tools to characterise emergence capacity in the human brain, in the context of functional and structural changes induced by severe brain injury. Bringing the notion of emergence from the realm of philosophy into neuroscience, we identified links between emergence and the spatio-temporal hierarchy of local-global interactions in the human brain, and further discovered a fundamental role of the structural connectome in supporting emergent and hierarchical dynamics. Taken together, the present results lead us to speculate that the chronic nature of unconsciousness in DOC patients may be due to permanent impairment of the fundamental neural infrastructures required to support hierarchical brain dynamics, capable of balancing local segregation and global integration - and ultimately the emergence of consciousness.

## 4 Methods

### 4.1 Disorders of consciousness patient data

The DOC patient data employed in this study have been published before (Luppi et al., 2019, 2022b; Spindler et al., 2021; Varley et al., 2020). For clarity and consistency of reporting, where applicable we use the same wording as our previous studies.

#### 4.1.1 Recruitment

As previously reported (Luppi et al., 2019), 71 DOC patients were recruited from specialised long-term care centres from January 2010 to December 2015. Ethical approval for this study was provided by the National Research Ethics Service (National Health Service, UK; LREC reference 99/391). Patients were eligible to be recruited in the study if they had a diagnosis of chronic disorder of consciousness, provided that written informed consent to participation was provided by their legal representative, and provided that the patients could be transported to Addenbrooke's Hospital (Cambridge, UK). The exclusion criteria included any medical condition that made it unsafe for the patient to participate, according to clinical personnel blinded to the specific aims of the study; or any reason that made a patient unsuitable to enter the MRI scanner environment (e.g., non-MRI-safe implants). Patients were also excluded based on significant pre-existing mental health problems, or insufficient fluency in the English language prior to their injury. After admission to Addenbrooke's Hospital, each patient underwent clinical and neuroimaging testing, spending a total of five days in the hospital (including arrival and departure days). Neuroimaging scanning took place at the Wolfson Brain Imaging Centre (Addenbrooke's Hospital, Cambridge, UK), and medication prescribed to each patient was maintained during scanning.

For each day of admission, Coma Recovery Scale-Revised (CRS-R) assessments were recorded at least daily. Patients whose behavioural responses were not indicative of awareness at any time, were classified as UWS. In contrast, patients were classified as being in a minimally conscious state (MCS) if they provided behavioural evidence of simple automatic motor reactions (e.g., scratching, pulling the bed sheet), visual fixation and pursuit, or localisation to noxious stimulation. Since this study focused on whole-brain properties, coverage of most of the brain was required, and we followed the same criteria as in our previous studies (Luppi et al., 2019, 2022b): before analysis took place, patients were systematically excluded if an expert neuroanatomist blinded to diagnosis judged that they displayed excessive focal brain damage (over one third of one hemisphere), or if brain damage led to suboptimal segmentation and normalisation, or due to excessive head motion in the MRI scanner (exceeding 3 mm translation or 3° rotation). A total of 22 adults (14 males; 17–70 years; mean time post injury: 13 months) meeting diagnostic criteria for unresponsive wakefulness syndrome/vegetative state (UWS; *N* = 10) or minimally conscious state (MCS; *N* = 12) due to brain injury were included in this study (Table 1). One patient only had functional data due to incomplete DWI acquisition.

**Table 1:** Demographic information for patients with Disorders of Consciousness.

Sex	Age	Aetiology	Diagnosis	CRS-R Score	Scan
M	46	TBI	UWS	6	12 dir
M	57	TBI	MCS	12	12 dir
M	46	TBI	MCS	10	Not available
M	35	Anoxic	UWS	8	12 dir
M	17	Anoxic	UWS	8	12 dir
F	31	Anoxic	MCS	10	12 dir
F	38	TBI	MCS	11	12 dir
M	29	TBI	MCS	10	63 dir
M	23	TBI	MCS	7	63 dir
F	70	Cerebral bleed	MCS	9	63 dir
F	30	Anoxic	MCS	9	63 dir
F	36	Anoxic	UWS	8	63 dir
M	22	Anoxic	UWS	7	63 dir
M	40	Anoxic	UWS	7	63 dir
F	62	Anoxic	UWS	7	63 dir
M	46	Anoxic	UWS	5	63 dir
M	21	TBI	MCS	11	63 dir
M	67	TBI	MCS	11	63 dir
F	55	Hypoxia	UWS	7	63 dir
M	28	TBI	MCS	8	63 dir
M	22	TBI	MCS	10	63 dir
F	28	ADEM	UWS	6	63 dir

CRS-R, Coma Recovery Scale-Revised; UWS, Unresponsive Wakefulness Syndrome; MCS, Minimally Conscious State; TBI, Traumatic Brain Injury.

#### 4.1.2 FMRI data acquisition

As previously reported (Luppi et al., 2019, (Luppi et al., 2021b), 2022b), resting-state fMRI was acquired for 10 min (300 vol, TR=2000 ms) using a Siemens Trio 3T scanner (Erlangen, Germany). Functional images (32 slices) were acquired using an echo planar sequence, with the following parameters: 3 × 3 × 3.75 mm resolution, TR = 2000 ms, TE = 30 ms, 78° FA. Anatomical scanning was also performed, acquiring high-resolution T1-weighted images with an MPRAGE sequence, using the following parameters: TR = 2300 ms, TE = 2.47 ms, 150 slices, resolution 1 × 1 × 1 mm.

#### 4.1.3 Acquisition of diffusion-weighted imaging data

As we previously reported (,(Luppi et al., 2021b) 2022b), the DOC patients’ data were acquired over the course of several years, and as a result two different diffusion-weighted image acquisition schemes were used. The first acquisition scheme involved diffusion-sensitising gradients applied along 12 non-collinear directions, and 5 different b-values ranging from 340 to 1590 s/mm^2^. An echo planar sequence was used (TR = 8300 ms, TE = 98 ms, matrix size = 96 × 96, 63 slices, slice thickness = 2 mm, no gap, flip angle = 90°). This acquisition scheme was used for the first *N* = 6 patients (Table 1). The second acquisition scheme included 63 directions with a b-value of 1000 s/mm2; this acquisition scheme was adopted for all remaining DOC patients and also for all healthy controls. Each of these DWI acquisition types has been used before with DOC patients ((Luppi et al., 2021b); 2022b; Wang et al., 2018; Zheng et al., 2017).

### 4.2 Healthy controls

We also used previously-acquired fMRI and DWI data from *N* = 20 healthy volunteers (13 males; 19–57 years), with no history of psychiatric or neurological disorders . The mean age was not significantly different between healthy controls (*M* = 35.75; SD = 11.42) and DOC patients (*M* = 38.24; SD = 15.96) (*t*(39) = −0.57, *p* = 0.571, Hedges's *g* = −0.18; permutation-based *t*-test).

#### 4.2.1 FMRI data acquisition

Resting-state fMRI was acquired for 5:20 min (160 vol, TR=2000 ms) using a Siemens Trio 3T scanner (Erlangen, Germany). The acquisition parameters were the same as those for the DOC patients: Functional images (32 slices) were acquired using an echo planar sequence, with the following parameters: 3 × 3 × 3.75 mm resolution, TR = 2000 ms, TE = 30 ms, 78° FA. High-resolution T1-weighted anatomical images were also acquired, using an MPRAGE sequence with the following parameters: TR = 2300 ms, TE = 2.47 ms, 150 slices, resolution 1 × 1 × 1 mm. Data from two subjects were excluded due to incomplete acquisition, leaving *N* = 18 healthy controls for the functional analysis.

#### 4.2.2 Acquisition of diffusion-weighted imaging data

The diffusion-weighted acquisition scheme was the same 63-directions scheme used for the DOC patients, as described above and in previous work (Luppi et al., 2021b): TR = 8300 ms, TE = 98 ms, matrix size = 96 × 96, 63 slices, slice thickness = 2 mm, no gap, flip angle = 90°, 63 directions with a b-value of 1000s/mm2.

### 4.3 Data preprocessing and denoising

#### 4.3.1 Functional MRI data

We preprocessed the functional imaging data using the CONN toolbox, version 17f (http://www.nitrc.org/projects/conn) (Whitfield-Gabrieli and Nieto-Castanon, 2012) based on Statistical Parametric Mapping 12 (http://www.fil.ion.ucl.ac.uk/spm). For each dataset and condition, we applied a standard preprocessing pipeline, the same as we employed in our previous studies (Luppi et al., 2019, 2020b, 2021c). The pipeline involved the following steps: removal of the first five volumes, to achieve steady-state magnetization; motion correction; slice-timing correction; identification of outlier volumes for subsequent scrubbing by means of the quality assurance/artefact rejection software art (http://www.nitrc.org/projects/artefact_detect); normalisation to Montreal Neurological Institute (MNI-152) standard space (2 mm isotropic resampling resolution), using the segmented grey matter image from each volunteer's T1-weighted anatomical image, together with an *a priori* grey matter template. Due to the presence of deformations caused by brain injury in the DOC patients, our preprocessing avoided automated pipelines. Each patient's brain was individually preprocessed using SPM12, with visual inspections after each step. Additionally, to further reduce potential movement artefacts, data underwent despiking with a hyperbolic tangent squashing function. These procedures are the same as in our previous publications on these data (Luppi et al., 2019, 2020b, 2022b).

To reduce noise due to cardiac and motion artefacts, we applied the anatomical CompCor method of denoising the functional data (Behzadi et al., 2007). The anatomical CompCor method (also implemented within the CONN toolbox) involves regressing out of the functional data the following confounding effects: the first five principal components attributable to each individual's white matter signal, and the first five components attributable to individual cerebrospinal fluid (CSF) signal; six subject-specific realignment parameters (three translations and three rotations) as well as their first- order temporal derivatives; the artefacts identified by *art;* and main effect of scanning condition. Linear detrending was also applied, and the subject-specific denoised BOLD signal timeseries were band-pass filtered to eliminate both low-frequency drift effects and high-frequency noise, thus retaining frequencies between 0.008 and 0.09 Hz. The step of global signal regression (GSR) has received substantial attention in the literature as a denoising method (Andellini et al., 2015; Lydon-Staley et al., 2019; Power et al., 2014). GSR mathematically mandates that approximately 50% of correlations between regions will be negative (Murphy and Fox, 2017); however, the proportion of anticorrelations between brain regions has been shown to vary across states of consciousness, including anaesthesia and DOC (Luppi et al., 2021b, 2022b; Tournier et al., 2019). Indeed, recent work has demonstrated that the global signal contains information about pathological and pharmacological states of unconsciousness (Tanabe et al., 2020). Therefore, in line with our previous studies, here we decided to avoid GSR in favour of the aCompCor denoising procedure, which is amongst those recommended for investigations of brain dynamics (Lydon-Staley et al., 2019).

#### 4.3.2 DWI preprocessing and tractography

The diffusion data were preprocessed with MRtrix3 tools, following the same pipeline as in our previous work (2022b; Tournier et al., 2019). After manually removing diffusion-weighted volumes with substantial distortion (Zheng et al., 2017), the pipeline involved the following steps: (i) DWI data denoising by exploiting data redundancy in the PCA domain (Veraart et al., 2016) (*dwidenoise* command); (ii) correction for distortions induced by eddy currents and subject motion by registering all DWIs to b0, using FSL's *eddy* tool (through MRtrix3 *dwipreproc* command); (iii) rotation of the diffusion gradient vectors to account for subject motion estimated by *eddy* (Leemans and Jones, 2009)*;* (iv) b1 field inhomogeneity correction for DWI volumes (*dwibiascorrect* command); and (v) generation of a brain masque through a combination of MRtrix3 *dwi2mask* and FSL *BET* commands.

After preprocessing, the DTI data were reconstructed using the model-free q-space diffeomorphic reconstruction algorithm (QSDR) implemented in DSI Studio (www.dsi-studio.labsolver.org) (Yeh et al., 2011), following our previous work (2022b; Luppi and Stamatakis, 2021). Use of QSDR is desirable when investigating group differences (Tan et al., 2019; Yeh et al., 2013, 2011) because this algorithm preserves the continuity of fibre geometry for subsequent tracking (Yeh et al., 2011), since it reconstructs the distribution of the density of diffusing water in standard space. This approach has therefore been adopted in previous connectomics studies focusing on healthy individuals (Gu et al., 2015) but also brain-injured patients (Gu et al., 2017) and DOC patients specifically (Tan et al., 2019; Yeh et al., 2013, 2011). QSDR initially reconstructs DWI data in native space, and subsequently computes values of quantitative anisotropy (QA) in each voxel, based on which DSI Studio performs a nonlinear warp from native space to a template QA volume in Montreal Neurological Institute (MNI) space. Once in MNI standard space, spin density functions are reconstructed, with a mean diffusion distance of 1.25 mm with three fibre orientations per voxel (Yeh et al., 2011).

Finally, fibre tracking was carried out by means of DSI Studio's own “FACT” deterministic tractography algorithm, requesting 1000,000 streamlines according to widely adopted parameters (Gu et al., 2017, 2015; 2022b): angular cutoff = 55°, step size = 1.0 mm, tract length between 10 mm (minimum) and 400 mm (maximum), no spin density function smoothing, and QA threshold determined by DWI signal in the cerebro-spinal fluid. Streamlines were automatically rejected if they presented improper termination locations, based on a white matter masque automatically generated by applying a default anisotropy threshold of 0.6 Otsu's threshold to the anisotropy values of the spin density function (Gu et al., 2017, 2015; 2022b).

### 4.4 Brain parcellation

For both BOLD and DWI data, brains were parcellated into 234 cortical and subcortical regions of interest (ROIs), according to the Lausanne sub-parcellation of the Desikan-Killiany anatomical atlas (Cammoun et al., 2012; Desikan et al., 2006). This parcellation has been used in previous work on controllability of structural brain networks (Gu et al., 2015; Tang et al., 2020). Recent work has shown that parcellations in the range of 200 regions provide generalisable network results (Luppi and Stamatakis, 2021). Additionally, to ensure the robustness of our results, we also replicated our analyses with a different version of the same parcellation, which includes 129 cortical and subcortical regions (Cammoun et al., 2012).

### 4.5 Quantifying emergence capacity

Consider a stochastic process X comprised of two random variables evolving jointly over time, $X_{t}=\left\{X_{t}^{1},\;X_{t}^{2}\right\}$. In our case, this corresponds to the timeseries of the BOLD activity of two brain regions, although in other applications it could be any form of multivariate timeseries data. One can now consider the amount of information flowing from the system's past to its future, known as time-delayed mutual information (TDMI) and given by $I(X_{t-\tau }^{1},X_{t-\tau }^{2};\;X_{t}^{1},X_{t}^{2})$ (Mediano et al., 2021).

Following the insights of Williams and Beer (2010), the information that two source variables *X* and *Y* give about a third target variable *Z*, denoted by *I*(*X,Y; Z*), can be decomposed in terms of different *types* of information: information provided by one source but not the other (unique information), by both sources separately (redundant information), or jointly by their combination (synergistic information). The mathematical framework of Integrated Information Decomposition (ΦID) (Mediano et al., 2021) has generalized this insight to the case of multiple sources and multiple target variables - such as the respective future states of the parts of the system under consideration. Thus, through ΦID it is possible to decompose TDMI into redundant, unique, and synergistic information shared with respect to both past and present state of both variables.

Importantly, ΦID's decomposition of information offers a way to compute the formal, quantitative definition of causal emergence established by Rosas and colleagues (Rosas et al., 2020), according to which a supervenient feature $V_{t}$ of system *X* is *causally emergent* if it has predictive power about the future evolution of $X_{t}$ that is unique with respect to $X_{t}^{1}$, …, $X_{t}^{n}$. Here, *supervenience* of $V_{t}$ on $X_{t}$ (the instantaneous state of the system at time *t*) is defined as $V_{t}$ being a function of $X_{t}$, so that there is nothing about $V_{t}$ that can be predicted from the system's previous state, $X_{t-1}$, that cannot be already predicted from the system's current state, $X_{t}$ (Rosas et al., 2020). Crucially, it can be mathematically demonstrated (Rosas et al., 2020) that a system's capacity for having causally emergent features depends directly on how synergistic its dynamics are. In particular, from ΦID's characterisation of causal emergence it follows that causal emergence can take place in two distinct scenarios: when an emergent feature has unique predictive power over parts of the system (“*downward causation*”), or when the emergent feature's unique predictive power is not over any individual constituent but only over the system as a whole (“*causal decoupling*”). The latter can be thought of as “the macroscale having causal influence on the macroscale, above and beyond the microscale effects” (Rosas et al., 2020). Here we focus on the system's “emergence capacity”, the combination of both downward causation and causal decoupling.

Conceptually, obtaining downward causation and causal decoupling involves obtaining the full integrated information decomposition of the system, which is achieved by setting up a linear system of 15 equations with 16 unknowns relating various standard (Shannon) mutual information terms with the redundant, unique, and synergistic components of the TDMI. The system can be solved by specifying the redundancy between $X_{t-\tau }$ and $X_{t}$. This provides a solution to the linear system of equations, from which all information atoms can be computed, and in turn downward causation and causal decoupling can be obtained as the sum of their constituent atoms. Practically, however, since our interest is in the aggregate of several atoms rather than individual atoms, causal emergence can also be computed more directly from partial information decomposition tools (https://github.com/robince/partial-info-decomp). Here, we follow the “common change in surprisal” (CCS) method (Ince, 2017). For all the analyses in the paper we compute information-theoretic quantities for each pair of brain regions, using a standard plug-in estimator applied to the mean-binarised BOLD signals. To validate our results, we also replicated them using continuous instead of discrete signals and the Gaussian solver implemented in the JIDT toolbox (Lizier, 2014). Likewise, we replicate our results using an alternative definition of redundancy known as the minimum mutual information (MMI) (Williams and Beer, 2010). In accordance with our previous work (Luppi et al., 2022c, 2020a) and previous studies using information-theoretic measures in the context of functional MRI data, for these analyses we used a state-of-the-art toolbox (Wu et al., 2013) to deconvolve the hemodynamic response function from our regional BOLD signal timeseries.

### 4.6 Spatiotemporal hierarchy from intrinsic-driven ignition

“Intrinsic-driven ignition” (Deco and Kringelbach, 2017) quantifies the extent to which spontaneously occurring (“intrinsic”) local events elicit whole-brain activation (“ignition”). For this analysis, first the BOLD signal is narrowband-filtered in the range 0.04–0.07 Hz range, in line with previous ^1^. The filtered timeseries are then transformed into z-scores, and subsequently thresholded to obtain a binary sequence σ based on the combined mean and standard deviation of the regional transformed signal, such that σ(t) = 1 if z(t) > 1 and is crossing the threshold from below, indicating that a local event has been triggered; otherwise, σ(t) = 0 ^1.^ Note that the threshold of 1 standard deviation for triggering an event is chosen for consistency with previous work, but it has been demonstrated that the results of this procedure are robust to the specific threshold chosen (Tagliazucchi et al., 2012). Subsequently, for each brain region, when that region triggers a local event (σ(t) = 1, (“driver event” in Fig. 2A), the resulting global ignition is computed within a time-window of 4 TRs (corresponding approximately to the duration of one hemodynamic response function, given our TR of 2 s). An NxN binary matrix M is then constructed (Fig. 2A), indicating whether in the period of time under consideration two regions *i* and *j* both triggered an event (M*_ij_ =* 1). The size of the largest connected component of this binary matrix M defines the breadth of the global ignition generated by the driver region at time *t,* termed “intrinsic-driven ignition” (IDI) (Deco and Kringelbach, 2017) (Fig. 2A). To obtain a measure of spatio-temporal hierarchy of local-global integration, each region's IDI values are averaged over time, and the variability (standard deviation) across regions is then computed. Consequently, higher standard deviation reflects more heterogeneity across brain regions with respect to their capability to induce ignition, which suggests in turn a more elaborate hierarchical organisation between them (Fig. 2A).

### 4.7 Structural network construction

A network consists of two basic elements: nodes, and the edges connecting them. To construct structural brain networks, patients’ brains were parcellated into 234 or 129 cortical and subcortical regions of interest (ROIs) of approximately equal size, derived from the Lausanne atlas (the parcels were dilated by 2 voxels to extend them to the grey-matter-white matter interface) (Gu et al., 2015). These ROIs represent the nodes of the brain network. Then, for each pair of nodes *i* and *j*, an edge was drawn between them if there were white matter tracts connecting the corresponding brain regions end-to-end; edge weights were quantified as the number of streamlines connecting each pair of regions end-to-end. In turn, this network can be represented as an adjacency matrix A, whose entry A_ij_ corresponds to the weight of connection between nodes (brain regions) *i* and *j*.

### 4.8 Network controllability

The model of brain dynamics used for network controllability analysis is based on extensive prior work demonstrating its wide applicability in health and disease (Betzel et al., 2016; Cornblath et al., 2018; Gu et al., 2015; Kim et al., 2018; Lynn and Bassett, 2019; Medaglia et al., 2017; Parker Singleton et al., 2021; Tang et al., 2020, 2017; Zarkali et al., 2020). In effect, there exists substantial evidence that linear models provide an adequate description of the brain dynamics measured with fMRI - such that more complicated non-linear models only capture little additional variance (Nozari et al., 2020; Schulz et al., 2020). Additionally, the controllability framework adopted here has been shown to have substantial overlap with the analysis of systems of non-linear oscillators connected with neurobiologically realistic coupling constants (using white matter connectivity, analogous to the use of white matter connectivity employed here) (Menara et al., 2021). Based on this literature and the well-known tractability of linear models, here we follow prior work on network control theory applications to structural brain networks (Gu et al., 2017, 2015) which uses a time-invariant network model on discrete-time of the form

(1) $$x\left(t+1\right)=Ax\left(t\right)+\;B_{K}u_{K}\left(t\right)$$

which has been previously used as a model of both BOLD signals and neural activity (Gu et al., 2015; Honey et al., 2009). Here, x is a vector describing the state of each brain region at a given point in time (e.g. in terms of neural activation as given by the BOLD signal magnitude - though note that the network control framework is agnostic about the nature of the system's activity), and A is the adjacency matrix representing the structural connectome (to ensure Schur stability, the adjacency matrix is divided by its largest singular value + 1 (Gu et al., 2017, 2015)). In turn, the input u_k_ represents the control strategy, which is applied according to the control points K identified by the matrix B_K_, where *K* = {*k_1_…k_m_*} and B_K_ = [$e_{k_{1}}$, … $e_{k_{m}}$] with *e_i_* representing the *i*th canonical vector of size N. While this is a time-discrete model, previous work has shown that the controllability Gramian (see below) is statistically similar to that obtained from continuous-time system (Gu et al., 2017, 2015).

Network control analysis enables us to investigate the ability of each brain region to influence the brain's dynamics in different ways. Technically, the “controllability” of a dynamical system (such as the human brain) refers to the extent to which the state of the dynamical system in question can be driven towards a chosen target state by means of an external input. Based on well-known results from control theory, the system described in Eq. (1) is controllable from the control nodes *K* if the “controllability Gramian” matrix given by

(2) $$W_{K}=\sum_{\tau =0}^{\infty }A^{\tau }B_{K}B_{K}^{T}A^{\tau }$$

is invertible. Following previous work, the input nodes are chosen one at a time, so that the input matrix B_K_ reduces to a vector denoting the control node.

Based on this controllability framework, we focus on two complementary control strategies for determining how the system can be moved towards different states (i.e., regional activation patterns): “average” and “modal” controllability (Gu et al., 2015).

#### 4.8.1 Average controllability

If the states that are accessible to the system are conceptualized as constituting an energy landscape, then average controllability describes how easily the system can transition between nearby states on this landscape. Average controllability of a network then equals to the average input energy needed at a set of control nodes, averaged over all possible target states. It is well-established that average input energy is proportional to $\text{Trace}(W_{K}^{-1})$. However, since the trace of the inverse Gramian is often uncomputable due to ill-conditioning, we follow previous work (Gu et al., 2015) in using $\text{Trace}(W_{K})\;$instead (which encodes the energy of the network impulse response), since the traces of the Gramian and its inverse are inversely proportional.

#### 4.8.2 Modal controllability

Modal controllability describes how easily the system can be induced to transition to a state that is distant on the energy landscape of its possible state. Technically, it corresponds to the ability of a node to control each of the dynamic modes of the network, and it can be computed from the matrix V of the eigenvectors of A. From well-established results, it is known that if the entry *v_ij_* is small, then the *j*th mode of the system is poorly controllable from node *i* (Gu et al., 2015). Therefore, here we follow previous work in defining a scaled measure of the controllability from brain region *i* of all the N modes of the system, $\lambda_{1}\left(A\right)$ … $\lambda_{N}\left(A\right)$ as:

(3) $$\varphi_{i}=\;\sum_{j=1}^{N}\left(1-\lambda_{j}^{2}\left(A\right)\right)v_{ij}^{2}).$$

From this definition, a region will have high modal controllability if it is able to control all the dynamic modes of the system, which implies that they are well-suited to drive the system towards difficult-to-reach configurations in the energy landscape (Gu et al., 2015).

For both average and modal controllability, whole-brain values can be obtained by taking the mean of all regional controllability values, as per prior work (Tang et al., 2017).

### 4.9 Whole-brain computational modelling

Macroscale whole-brain computational models represent regional activity in terms of two key ingredients: (i) a biophysical model of each region's local dynamics; and (ii) inter-regional anatomical connectivity. Thus, such *in silico* models provide a well-suited tool to investigate how the structural connectivity of the brain shapes the corresponding macroscale neural dynamics (Cabral et al., 2017; Cofré et al., 2020; Deco and Kringelbach, 2014; Demirtaş et al., 2019; Kringelbach and Deco, 2020; Shine et al., 2021; Wang et al., 2019). In particular, the Dynamic Mean Field (DMF) model employed here simulates each region (defined via an anatomical parcellation scheme) as a macroscopic neural field comprising mutually coupled excitatory and inhibitory populations (80% excitatory and 20% inhibitory), providing a neurobiologically plausible account of regional neuronal firing rate. Regions are then connected according to empirical anatomical connectivity obtained e.g. from DWI data (Deco et al., 2014; G. 2013; Deco and Jirsa, 2012). The reader is referred to (Deco et al., 2018; Herzog et al., 2022; 2020; Luppi et al., 2022b) for details of the DMF model and its implementation. Due to its multi-platform compatibility, low memory usage, and high speed, we used the recently developed *FastDMF* library (Herzog et al., 2022), available online at https://www.gitlab.com/concog/fastdmf.

The structural connectivity (SC) for the DMF model used here was obtained by following the procedure described by Wang et al. (2019) to derive a consensus structural connectivity matrix. A consensus matrix *A* was obtained separately for each group (healthy controls, MCS patients, UWS patients) as follows: for each pair of regions *i* and *j*, if more than half of subjects had non-zero connection *i* and *j, A_ij_* was set to the average across all subjects with non-zero connections between *i* and *j*. Otherwise, *A_ij_* was set to zero.

The DMF model has one free parameter, known as “global coupling” and denoted by *G*, which accounts for differences in transmission between brain regions, considering the effects of neurotransmission but also synaptic plasticity mechanisms. Thus, separately for each group, we used a model informed by that group's consensus connectome to generate 40 simulations for each value of *G* between 0.1 and 2.5, using increments of 0.1. Finally, we set the *G* parameter to the value just before the one at which the simulated firing of each model became unstable, reflecting a near-critical regime.

Subsequently, for each group, 40 further simulations were obtained from the corresponding DMF model with the optimal *G* parameter. A Balloon-Windkessel hemodynamic model (Friston et al., 2003) was then used to turn simulated regional neuronal activity into simulated regional BOLD signal. Finally, simulated regional BOLD signal was bandpass filtered in the same range as the empirical data (0.008–0.09 Hz, or 0.04–0.07 Hz for the intrinsic ignition analysis).

As an alternative way of finding the most suitable value of *G* for the simulation of each condition, we adopted the approach previously described (Deco et al., 2018; Hansen et al., 2015; Herzog et al., 2020; Luppi et al., 2022b) which aims to obtain the best match between empirical and simulated functional connectivity dynamics. First, we quantified empirical functional connectivity dynamics (FCD) in terms of Pearson correlation between regional BOLD timeseries, computed within a sliding window of 30 TRs with increments of 3 TRs (Deco et al., 2018; Hansen et al., 2015; Herzog et al., 2020; Luppi et al., 2022b). Subsequently, the resulting matrices of functional connectivity at times *t_x_* and *t_y_* were themselves correlated, for each pair of timepoints *t_x_* and *t_y_*, thereby obtaining an FCD matrix of time-versus-time correlations. Thus, each entry in the FCD matrix represents the similarity between functional connectivity patterns at different points in time. This procedure was repeated for each subject of each group (controls, MCS, and UWS). For each simulation at each value of *G*, we used the Kolmogorov-Smirnov distance to compare the histograms of empirical (group-wise) and simulated FCD values (obtained from the upper triangular FCD matrix). Finally, we set the model's *G* parameter to the value that was observed to minimize the mean KS distance - corresponding to the model that is best capable of simulating the temporal dynamics of resting-state brain functional connectivity observed in the corresponding group (Figure S8). After having found the value of *G* for each condition, simulated BOLD signals were obtained as described above. This same procedure was also used for fitting the DMF model based on each individual's structural connectome, simulating BOLD signals to fit their own empirical FCD.

### 4.10 Statistical analysis

Statistical significance of differences in functional measures was assessed by conducting a three-way analysis of variance (ANOVA), testing for the effect of interest (diagnostic condition, with three levels: control, MCS and UWS). Upon finding the effect of interest to be statistically significant, we conducted post-hoc tests (two-sided non-parametric between-subjects t-tests with 10,000 permutations) using three pairwise comparisons between the conditions (control vs. MCS, control vs. UWS, and MCS vs. UWS). We adopted the method of Benjamini and Hochberg (1995) to control the false discovery rate across these three pairwise comparisons, at a two-sided alpha value of 0.05. The effect sizes were estimated using Cohen's *d*. For the statistical analysis of differences in structural measures (global average and modal controllability), we used an analysis of covariance to control for DWI sequence type (12 vs 63 directions) and the number of removed volumes due to motion corruption, as covariates of no interest (Luppi et al., 2021b). Although we thoroughly preprocessed our functional data to minimise the potential confounding effects of head motion, to ensure the robustness of our results we also carried out a validation analysis including motion (mean framewise displacement) as a covariate of no interest in the functional analyzes.

## 5 Data and code availability

We have made available multi-platform code for the FastDMF model at https://www.gitlab.com/concog/fastdmf. We also provide annotated MATLAB code to follow our workflow as Supplementary Material, including code for Information Decomposition implementing the measures used in this study.

Due to patient privacy concerns, data are available upon request by qualified researchers. The UK Health Research Authority mandates that the confidentiality of data is the responsibility of Chief Investigators for the initial studies (in this case, Dr. Allanson and Prof Menon; and anyone to whom this responsibility is handed – for example, in the context of retirement or transfer to another institution). For researchers interested in working with this dataset, please contact Dr. Judith Allanson (judith.allanson@addenbrookes.nhs.uk), Prof. David Menon (dkm13@cam.ac.uk) and/or Dr. Emmanuel Stamatakis (eas46@cam.ac.uk). Requests will be considered on a case-by-case basis, assessing the feasibility and appropriateness of the proposed study, and the capacity to maintain the required levels of data security, consistent with the original approved Research Ethics approval, and the patient information sheet that was the basis of consent obtained.

## CRediT authorship contribution statement

**Andrea I. Luppi:** Conceptualization, Methodology, Software, Validation, Formal analysis, Writing – original draft, Writing – review & editing, Visualization. **Pedro A.M. Mediano:** Conceptualization, Methodology, Software, Formal analysis, Writing – review & editing. **Fernando E. Rosas:** Conceptualization, Methodology, Formal analysis, Writing – review & editing. **Judith Allanson:** Conceptualization, Resources, Investigation, Data curation, Writing – review & editing, Project administration. **John D. Pickard:** Conceptualization, Resources, Writing – review & editing, Project administration, Funding acquisition. **Guy B. Williams:** Investigation, Conceptualization, Writing – review & editing. **Michael M. Craig:** Formal analysis, Data curation, Writing – review & editing. **Paola Finoia:** Investigation, Data curation. **Alexander R.D. Peattie:** Formal analysis, Writing – review & editing. **Peter Coppola:** Formal analysis, Writing – review & editing. **David K. Menon:** Conceptualization, Investigation, Resources, Writing – review & editing, Supervision, Project administration, Funding acquisition. **Daniel Bor:** Writing – review & editing, Supervision. **Emmanuel A. Stamatakis:** Conceptualization, Resources, Methodology, Investigation, Writing – original draft, Writing – review & editing, Supervision, Project administration, Funding acquisition.

## Declaration of Competing Interest

The authors declare no competing interests.

## Data Availability

Data will be made available on request.

## Appendix Supplementary materials

Image, application 1

Image, application 2
